# Structure-based Methods for Computational Protein Functional Site Prediction

**DOI:** 10.5936/csbj.201308005

**Published:** 2013-11-11

**Authors:** B KC Dukka

**Affiliations:** a Department of Computational Science and Engineering, North Carolina A&T State University, Greensboro, NC, 27411, USA

## Abstract

Due to the advent of high throughput sequencing techniques and structural genomic projects, the number of gene and protein sequences has been ever increasing. Computational methods to annotate these genes and proteins are even more indispensable. Proteins are important macromolecules and study of the function of proteins is an important problem in structural bioinformatics. This paper discusses a number of methods to predict protein functional site especially focusing on protein ligand binding site prediction. Initially, a short overview is presented on recent advances in methods for selection of homologous sequences. Furthermore, a few recent structural based approaches and sequence-and-structure based approaches for protein functional sites are discussed in details.

## Introduction

Proteins bind with other molecules to bolster or inhibit biological functions. In all these protein and the binding partner (ligand) interaction, usually a few key residues are involved. It is important to identify these key sites in order to understand the function of the protein. Most of the computational approaches to recognize these functional sites in proteins can broadly be classified into sequence or structure based methods. There have been some magnificent works [1] in regard to reviewing the existing tools and methods in this field. However, there have been a lot of progress and new types of methodology developed for protein functional site prediction. In this regard, this paper aims to briefly describe some of the recent developments in the field of protein functional site prediction for structure-based approaches and sequence-and-structure based approaches. Firstly, a brief overview of sequence-based approaches is presented along with some recent development. Then, a detailed overview on structure-based approaches and sequence-and-structure based approaches for protein functional site prediction is presented.

## Sequence based Approaches

The main strength of the sequence-based approaches for binding site prediction is that these methods have the ability to determine a ligand-binding motif in proteins that may not have same overall fold. However, if the binding site (pocket) is nonlocal or non-contiguous in sequence, motif based approach generally becomes ineffective. Homology-based methods require related proteins with significant identity to the query protein in the protein data bank (PDB) because the conservation of biochemical function drops rapidly for proteins sharing < 35-40% sequence identity [2]. Various sequence based approaches have been developed over the years including, but not limited to, Conseq [3] Conservation Scores [4], MINER [5–7] and so on. Refer to the excellent review on phylogenetic based approaches for more details about these methods [8].

Essentially, for methods based on sequence-based approaches, at first, the homologous sequences of a target sequence are collected and a multiple sequence alignment (MSA) is constructed. Then, using various approaches conserved residues are identified among all the sites in the MSA.

Selection of homologous sequences to a query protein is a critical step in all sequence-based and sequence and structure based approaches for protein functional site prediction. Despite the fact, this problem has not been sufficiently addressed. In this regard, how to select appropriate homologous sequences for the identification of conserved residue is a contentious issue. Some of the recent approaches to address this issue are discussed below.

## Appropriate Selection of Homologous Sequences

As mentioned earlier, selection of homologous sequences is critical step in sequence-based and sequence and structure based approaches for protein functional site prediction. It has been empirically shown that certain degree of sequence divergence is required in an MSA for the identification of functional sites. However, there is no concrete objective criterion and selection of sequences for the MSA is unavoidably subjective [9].

In this regard, Aloy et al. [10] developed an automatic method to predict the functional regions of a protein by using some criteria for selection of homologous sequences. Essentially, in their method, the clustering of the conserved residues on the tertiary structure is evaluated. If no cluster is identified, then the MSA is reconstructed by removing the distant homologues of the target protein. This process is iterated until the cluster of conserved residues is identified.

Mihalek et al. [11] also proposed ‘residue clustering measure’ to indicate the appropriateness of the homologous sequences for functional region prediction. This measure essentially quantifies the degree of clustering of the evolutionarily important residues in the tertiary structure of the protein. The measure assigns greater importance to the clustering of the residues that are far from each other on the primary sequence. The usefulness of the measure was proved by applying the measure to improve the performance of the real valued Evolutionary Trace (rvET) [12].

Recently, Nemoto et al. [9] developed a novel index to select the appropriate set of sequences for the identification of conserved residues, and implemented the index to show the usefulness of the index for the prediction of functional regions of a protein. The index is called DSPAC index.

## DSPAC Index

In order to select the appropriate set of sequences for functional regions prediction, Nemoto et al. [9] developed the DSPAC index. The DSPAC (Degree of SPatial Auto-Correlation) is quite often used in spatial statistics to detect local clusters. The full explanation of DSPAC is outside the scope of the review and interested readers are requested to refer to Nemoto et al. [9].

The DSPAC indicates the degree of spatial autocorrelation and is used as an index of the appropriateness of a set of homologous sequences. Precisely, a set of homologous sequences of a target protein is generated and divided into some subsets. Among the subsets, the subset with the maximum DSPAC is chosen. Finally, the set of sequences corresponding to the MSA that has the maximum DSPAC is adopted as the most appropriate set of sequences for the function region prediction of the query protein.

The usefulness of the index was demonstrated by improved performance of functional region prediction in the FREPS program. It has to be noted here that the index for selecting appropriate homologous sequences is called DSPAC and the method that uses this index is called FREPS. FREPS is discussed in sequence and structure based method section.

Next, we will discuss widely used methodologies and recent advances in structure-based approaches for functional site prediction.

## Structure based Approaches

Based on the observation of existing protein-ligand complexes, it is quite evident that homologous proteins with similar global topology often bind similar ligands using a conserved set of residues [13]. In this regard, there are various methods that utilize both geometric match and evolutionary information to identify binding site.

A number of structure-based approaches for binding site prediction have been developed. These methods are broadly classified into: geometry based approaches and energetic based approaches. Geometry-based approaches identify binding residues by searching for pockets/cavities in a protein structure. Energetic-based approaches identify binding residues by using various interaction energies. Recently, there have also been recent developments of structure alignment based methods for functional site prediction.

## Structure Alignment based Methods

Due to the availability of large number of solved protein structures in databases like Protein Data Bank [14] and development of sophisticated protein structure prediction protocols [15, 16], it is now possible to develop methods based on structure alignment of proteins. In this regard, there have been a couple of methods developed in this area. These methods may be broadly classified as global structure alignment based methods and local structure alignment based methods.

## Global Structure Alignment based Approaches

FINDSITE [17] is a threading-based approach for binding site prediction based on global structure alignment and was developed in the Skolnick Lab. FINDSITE's spirit is based on the observation, of systematic analysis of know protein structures grouped according to SCOP [18] classification, that there is a general tendency of certain protein folds to bind substrates at a similar location. This observation suggests that distantly homologous proteins can have common binding sites and if indeed that is the case it should be possible to identify ligand-binding sites for not so perfect structure (modeled structures).

Given a query protein, FINDSITE first identifies a group of template structures user threading. Essentially, PROSPECTOR_3 [19] threading algorithm identifies ligand-bound structural templates. Also, based on these threading templates, a model of the query protein is generated. Then, these holo-templates are superimposed onto the predicted target protein structure by TM-align structure alignment algorithm. The clustered centers of mass of the ligands bound to the threading templates identify putative binding sites and the predicted sites are ranked according to the number of templates that share a common binding site.

## Local structural alignment based Approaches

Local alignment approaches are suited to detect locally conserved patterns of functional groups, which often appear in binding sites and have significant involvement in ligand binding. In this regard, ProBis[20] enables the local structural alignment of entire protein surface structure against a large database of protein structures and then detects structurally similar regions in a query protein by mapping structural similarity scores on its surface.

ProBis is a local structure alignment (LSA) based algorithm developed at Janezic's lab to detect locally similar surface patches of proteins independent of the protein fold. The algorithm identifies structurally similar sites, whose residues may be scattered in the sequence space, but are close together in structure. Such patches are often related to ligand binding sites and searching for these sites exploits the fact that protein performing similar functions may share similar patterns of interactions of binding sites. ProBis uses a graph theoretical approach (clique based approach) by constructing protein graphs. Clique based algorithms have been previously applied to protein side-chain packing problems [21].

Initially, protein surface residues are identified by calculating the solvent accessible surface atoms of the protein. The surface amino acid residues are then assigned to one of the five labels based on the physicochemical properties of the functional groups: hydrogen-bond donor, hydrogen-bond acceptor, mixed acceptor/donor, aromatic, and aliphatic groups. Each functional group is then substituted with one labeled point that represents potential interactions of this particular functional group with other molecules. Essentially, the vertices correspond to the functional groups of surface amino acid residues and the distance between pairs of adjacent vertices determines the edges. In this regard, this representation captures both geometric and physiochemical properties. Finally, different protein surfaces are compared by constructing protein product graphs, followed by a search for maximum cliques in these graphs. These maximum cliques in the product graphs correspond to protein surface similarities.

The product graph obtained from the sub-graphs is then subjected to maximum clique finding algorithm and a maximum clique corresponds to a largest common vertex substructure. Alignment scores like surface vector angle, RMSD and E-values are calculated for each local superimposition. It is important to note that each of these pairwise comparisons may result in a number of different maximum cliques where clique represents the local structural similarities between the compared proteins. Finally, a search for similarities in flexible parts of the two compared proteins is conducted (Detail omitted here).

In essence, ProBiS conducts searches for similar 3D structural regions in proteins without reference to known binding sites or co-crystallized ligands taking into account entire protein surfaces.

## Global and Local structure alignment based approaches

COFACTOR [22] is a method that falls in the category of global and local structure alignment based approaches. This is a method to predict binding site residues in a protein. It starts with a query sequence and then a 3D structure model is generated for the query sequence using I-TASSER [15] algorithm. Based on the global structural similarity to the query protein using the TM-align [23] structure alignment program, template proteins with bound ligands in the PDB [14] are collected.

Meanwhile, the binding pockets of template structures are scanned through the query protein structure to identify the best local geometric and sequence matches. The binding pose of the ligand in the query structure is predicted based on the local alignment of predicted and template binding site residues.

The major difference between COFACTOR and the existing methods is the combination of global and local structural comparisons for identifying ligand-binding sites. It generally outperforms other cavity-based methods when only low-resolution protein models are available because the global topology comparisons can reliably identify the correct functional template. Furthermore, for proteins that have functional templates with different global topology but similar conserved binding pockets, the local structural alignment inside COFACTOR helps to recognize the ligand-binding residues unlike purely global structural comparison methods like FINDSITE [17].

## Geometric Based Methods

As discussed earlier, besides the structure alignment based methods, there are methods based on geometry. In this section, we will review some of the widely used geometric based approaches for protein ligand-binding site prediction.

### POCKET

POCKET [24] algorithm introduced the idea of protein-solvent-protein events as the key concept for identification of binding sites. The protein is mapped onto a 3D grid. A grid point is a part of the protein if it is within 3A of an atom coordinate; otherwise it is a solvent. Next, the x-, y-, and z-axes are scanned for the pockets, which are characterized as a sequence of grid points, which start and end with the label protein and having a period of solvent grid points in between. These sequences are called protein-solvent-protein events and only grid points that exceed a threshold of protein-solvent-protein events are retained for the final pocket prediction. Here we discuss, some of the ligand binding site prediction methods that analyze the protein surface for pockets. The ligand binding site is usually in the largest pocket.

### LIGSITE

LIGSITE [25] is a method for predicting protein binding sites using the Connolly Surface and conservation. LIGSITE extends POCKET by scanning along the four cubic diagonals in addition to the x, y, and z directions. Furthermore, two extensions were introduced to LIGSITE: First, instead of capturing protein-solvent-protein events, the more accurate surface-solvent-surface events using the protein's Connolly surface is captured and this extension is called LIGSITE^cs^ (cs= Connolly surface). Second, the pockets identified by the surface-solvent-surface events are re-ranked by the degree of conservation of the involved surface residues and this extension is called LIGSITE^csc^ [26] (csc = Connolly surface and conservation).

LIGSITE^csc^ is an extension of LIGSITE. Instead of defining protein-solvent-protein events on the basis of atom coordinates, it uses the Connolly surface and defines surface-solvent-surface events. The steps in the algorithm are as follows. First, the protein is projected onto a 3D grid. For the grid, a step size of 1.0Å is used. Then, the grid points are labeled as protein, surface, or solvent based on following rules:

- A grid point is a protein if there is at least one atom within 1.6Å.
- Solvent excluded surface is calculated using the Connolly algorithm and the surface vertices’ coordinates are stored. A grid point is marked as surface if a surface vertex is within 1.0Å.
- All other grid points are labeled as solvent.

Finally, a sequence of grid points, which starts and ends with surface grid points and which has solvent grid points in between is called surface-solvent-surface event. LIGSITE^csc^ scans the x, y, and z directions along with four cubic diagonals for such surface-solvent-surface events. If the number of these types of events exceeds 6, the grid is marked as ‘pocket’. Finally, if a pocket grid point is within 3.0Å to a pocket grid point cluster, it is added to the cluster or else it is considered a new cluster. Next, the clusters are ranked by the number of grid points in them. The top three clusters are retained and their centers of mass are used to represent the predicted ‘pocket sites’. Finally, the top 3 pocket sites are re-ranked according to conservation score. The conservation score of the involved surface residues is the average conservation score of all residues within a sphere of 8A radius from the center of the mass of the cluster.

### SURFNET

The SURFNET [27] algorithm identifies the clefts on a protein surface by placing a sphere between all pairs of atoms such that sphere just touches each atom and is between some predefined minimum and maximum radius.

Each sphere is reduced in size if any other atoms intersect it until: i) it intersects with no further atoms or ii) its radius drops below the minimum size. If it intersects with no further atoms the sphere is retained else the sphere is discarded. Once, the clefts on the surface have been filled by spheres, it is possible to cluster the spheres into separate regions and calculate a volume for each cleft. The approach is particularly useful for locating binding sites in proteins.

## Energetic-based Approaches

As discussed earlier, besides geometric based approaches, there are a number of energetic based approaches. Some of the energetic-based approaches are discussed below.

## Q-SiteFinder

Q-SiteFinder [28] is a ligand binding site prediction tool. It is an energetic based approach to find ligand binding pockets. In Q-SiteFinder, the protein surface is coated with a layer of methyl (-CH3) probes to calculate van der Waals interaction energies between the protein and probes. The probes with most favorable interaction energies are retained. The probe coordinates are saved and the coordinates are rotated back to match the original orientation of the protein.

Individual probe coordinates are then clustered according to the spatial proximity and the total interaction energies of probes within each cluster is calculated. The probe clusters are ranked according to their total interaction energies and the cluster with the most favorable is identified as the binding sites.

A recent version of Q-SiteFinder [29] achieves a higher success rate by using a better probe distribution technique and more suitable force field parameters to calculate interaction energies.

## Pocket-Finder

Pocket-Finder is a tool for identifying protein-ligand binding sites. Pocket-Finder implements LIGSITE. Like, LIGSITE Pocket-Finder measures the extent to which each grid point is buried in the protein. Each grid point has seven scanning lines passing through it (in the x, y and z directions plus the four cubic diagonals). The grid points are initially set to zero. These points can have a value from zero (not part of a pocket) to seven (deep in the cavity) protein-site-protein event. Details are omitted here. Using a grid resolution of 0.9Å and a probe radius of 1.6Å and the threshold number of PSP events of 5, pockets are defined by cubes of retained grid points.

The major difference between Pocket-Finder and Q-SiteFinder is that in clustering in Pocket-Finder the sites produced by the Pocket-Finder are ranked according to the number of probes in the site rather than by probe energy. Another difference between Q-SiteFinder and Pocket-Finder is the value of parameter for estimation of site volume. For Q-SiteFinder a value of 5.0Å is used and for Pocket-Finder a value of 3.0Å is used. These values reflect the fact that the probe site identified in Q-SiteFinder approach the protein within van der Waals (vdW) contact whereas sites approach the vdW surface of proteins in terms of Pocket-Finder.

## Fuzzy Oil Drop based Approach (Discuss more about FOD algorithm)

Various methods based on structural analysis coupled with surface hydrophobicity have been used to identify protein functional sites [30]. In this regard, Brylinski et al. [31] developed a method for protein functional site prediction based on the Fuzzy Oil Drop Model (FOD). The Fuzzy Oil Drop (FOD) model is based on an external hydrophobic force field.

The FOD hydrophobic force field is based on the assumption that the theoretical hydrophobicity distribution in proteins is represented by the 3-D Gaussian function. For this reason, these values can be considered equal to zero. The size of the molecule is expressed by the triple σx, σy, σz, which is calculated for each molecule individually provided that the orientation of the molecule with the longest possible inter-effective atoms distance is determined according to the appropriate coordinate system axis. The σ values are calculated as the 1/3 of the longest distance between two effective atoms calculated along each axis. The value of the Gauss function at any point of protein body is treated as the idealized hydrophobic density defining the hydrophobic core.

The idealized hydrophobicity at any point of the “fuzzy oil drop” can be calculated according to the Gauss function for the molecule located with its geometric center as the origin of the coordinate system.

The second component of this force field is an observed hydrophobicity distribution formed by the side chains of a protein molecule [32]. The scoring function is based on the difference between the theoretical and empirical distributions of hydrophobicity that expresses the irregularity of hydrophobic core construction. The maxima of the difference recognize the residues representing the hydrophobicity deficiency, which are normally function-related.

Essentially, the difference between the theoretical (expected) hydrophobicity and the observed values of hydrophobicity is used to characterize the functional sites. See [31] for the details. The maxima of the difference recognizes the residues representing the hydrophobicity deficiency, which in turn, points out to the structural irregularity that is usually in a function related area.

According to FOD, the hydrophobic residues tend to be placed in the central part of the protein molecule while hydrophilic residues on the protein's surface [33].

This method to predict functional sites is based on the observation that there is a high discrepancy between observed and theoretical hydrophobicities within FOD in the area of the binding sites.

The usefulness of the method for protein binding site detection was verified by comparing the method with SuMo [34] and ProFunc [35].

## FTSite

FTSite algorithm [36] is developed at Vajda Laboratory at Boston University and is an energy-based method. This method is mainly used for accurate detection of ligand binding sites on unbound protein structures. This method does not rely on any evolutionary or statistical information. It is based on experimental evidence that ligand-binding sites also bind small organic molecules of various shapes and polarity, as observed by Nucleic Magnetic Resonance (NMR) [37]. Based on this assumption, FTSite is based on a solvent mapping algorithm [38] which places each of 16 different small molecular probes on a dense grid around the protein and finds favorable positions using empirical free energy function. Then, for each probe type, the individual probes are clustered and the clusters are ranked based on the average free energy.

Subsequently, consensus clusters are identified as sites in which different probe clusters overlap. The consensus clusters are ranked based on the total number of non-bonded interactions between the protein and all probes in the cluster. The consensus cluster with the highest number of contacts is ranked first whereby nearby consensus clusters are also joined with the cluster. Finally, the amino acids in contact with the probes of the newly defined cluster are predicted to be the top rank ligand-binding site. The stark difference of FTSite compared with other energy-based methods is that it uses multiple molecular probes rather than a single probe.

## Sequence-and-Structure based Approach

As discussed above, there have been myriad of approaches to predict protein funcationl region based on sequence properties that have largely exploited sequence conservation or to accept fewer mutations relative to the overall protein. On the other hand, there are numerous approaches based on structural properties that have used geometric and energetic properties. In this regard, there have been various approaches to combine sequence and structure-based feature to improve protein functional site prediction. One of the seminal methods in this category is Evolutionary Trace[39].

## Concavity

In this regard, ConCavity [40] falls in the type of approach that tries to marry these two distinguishing approaches by integrating sequence conservation with structural properties to predict protein ligand binding sites. ConCavity developed at Singh's lab in Princeton is a protein ligand binding site prediction algorithm that integrates evolutionary sequence conservation with structure-based methods for identifying protein surface cavities.

Initially, a grid of points surrounding the protein surface is scored by combining the output of a structure based pocket finding algorithm, mainly, LIGSITE[25], SURFNET [27] or PocketFinder [28] with the sequence conservation values of nearby residues. Secondly, coherent pockets are extracted from the grid using 3D shape analysis algorithm to ensure that the predicted pockets have reasonable shapes and volumes. In the final step, the residues are mapped by assigning high scores to residues near high scoring pocket grid points. In essence, ConCavity algorithm consists of three major steps: grid creation, pocket extraction, and residue mapping.

1) Grid Creation

Grid creation is similar to other grid creation ligand binding site prediction approaches like POCKET [24] and LIGSITE. The structural and evolutionary properties of a given protein are used to create a regular 3D grid surrounding the protein in which the score associated with each grid point represents an estimated likelihood that it overlaps with a bound ligand atom.

Major difference between other grid based approaches and ConCavity is that ConCavity integrates evolutionary information directly into the grid creation step.

2) Pocket Extraction

The second step is to cluster groups of contiguous, high scoring grid points into pockets.

3) Mapping

Mapping residue is the third step in the pipeline and it uses the extracted set of pockets to generate ligand-binding predictions for residues.

In essence, ConCavity uses the grid creation methods of LIGSITE [25], SURFNET [27] and PocketFinder [28]. In addition to that, the evolutionary information is integrated in the grid creation process by weighing the ‘votes’ as the grid is created by an estimate of sequence conservation of the residue associated with the atoms that generate the votes. The conservation scores are calculated by the Jensen-Shannon divergence.

Comparison of ConCavity to several methods for ligand binding site prediction shows that ConCavity outperforms many of the existing methods for ligand binding site prediction.

## ConSurf

ConSurf [41] is a protein functional site prediction tool developed at Ben-Tal's Lab. ConSurf uses sequence of the query protein if the query is a sequence and it can also take 3D structure as an input and in this case, the sequence corresponding to the structure is extracted. ConSurf is also explained in detail in the following book chapter [8].

Given a sequence, initially, ConSurf searches for homologous sequences. As discussed earlier, selection of homologous sequence is subjective so ConSurf offers various choices for the uses. A MSA of the collected sequences is performed by MAFFT [42]or other methods and a phylogenetic tree is constructed based on the MSA. Finally, position specific conservation scores are calculated using the Rate4Site algorithm [43]. If the input is sequence, ConSurf projects the conservation grades on a related structure and if the input is a structure, it projects the conservation grades on the provided structure.

LIGSITE^csc^ [26] can also be classified as the method based on both sequence and structural features. However, strictly speaking as LIGSITE^csc^ only uses evolutionary conservation in a post-processing step to re-rank, hence we discussed LIGSITE^csc^ in the structure-based ligand site prediction section.

## SURFNET-ConSurf

The SURFNET-ConSurf [44] is a method for identifying ligand binding sites in proteins and falls on the category of the methods which try to marry structural properties with sequence conservation. In the first stage SURFNET [27] program identifies clefts in the protein surface that are potential binding sites. In the second stage, these clefts in the protein surface are trimmed in size by cutting away regions distant from highly conserved residues using ConSurf [45].

The SURFNET [27] algorithm identifies the clefts on a protein surface by placing a sphere between all pairs of atoms such that sphere just touches each atom and is between some predefined minimum and maximum radius.

The second part involves discarding the spheres that are distant from highly conserved residues. The residue conservation scores are obtained from ConSurf-HSSP database version 1 [46], which provides estimates for the rate of evolution of each amino acid in a PDB structure. For details of the ConSurf, see [45] or review paper [8]. While discarding the spheres, those spheres that are within a certain distance of any atom of the highly conserved residues are kept, and those that are not are discarded from the sphere list, thus reducing the original cleft volume. Two parameters: maximum allowed distance between the atom and the sphere and the minimum conservation score cutoff are optimized using calibration and the value of these parameters are chosen to be 3.5Å and a ConSurf cutoff of 8.

This method was tested on a set of 244 proteins and in 75% of the proteins the ligand -binding pocket was identified correctly. It should be noteworthy that even though for a few cases the ligand binding sites that were identified by SURFNET were totally lost after the conservation filtering stage, this method proves the concept that a simple combination of conservation and binding site volume provides a reasonable improvement in binding site prediction.

## FREPS

Functional Region Prediction of a protein by Spatial statistics (FREPS) [9] is a method to predict functional regions of a protein by using structure and homologous sequences of a target protein. The basic strategy of the method is to detect spatial clusters of conserved residues on the protein structure.

The method utilizes spatial statistics, which takes into account spatial position of the data. In addition, as discussed earlier, a novel DSPAC index is used to select the appropriate set of sequences.

Based on two assumptions, viz. i) most of the functional regions of proteins are exposed or at least semi-buried on the molecular surface and ii) the amino acid residues conserved among homologues are abundant at the functional regions, due to functional constraints, FREPS identifies functional regions of a protein by examining the cluster of conserved residues on the protein surface with spatial statistics techniques. Essentially, a modified Local Moran's I score [47] called as Local Moran's I by using conservation score (LMIC) is implemented to detect clusters of conserved residues. Interested readers are advised to refer to the FREPS paper [9] for the details.

## Meta Server Approaches

### MetaPocket

There are some approaches where existing algorithms are combined to get the consensus among these algorithms. One of them is MetaPocket 2.0 [48]. MetaPocket 2.0 is a meta-server which seeks consensus among 4 different methods and reaches 80% accuracy for the unbound LIGSITE^csc^ test set for prediction of ligand binding sites. MetaPocket combines together four protein binding site predictors and proposes a meta-method to predict ligand-binding sites. The 4 combined methods are: LIGSITE^csc^ [26], PASS [49], Q-SiteFinder [28], and SURFNET [27].

In essence, for each protein structure, four of the approaches mentioned above are used to identify pocket sites. It is not readily possible to combine or compare the results of these 4 methods as the pocket sites identified as these methods have different ranking scoring functions. Hence, in order to do that, a z-score is calculated separately for each site in different methods. Then, only the top three pocket sites in each method are taken into further consideration.

Hence, a total of 12 pocket sites (3 from each of the 4 methods) are obtained and these sites are clustered using hierarchical clustering algorithm. Probes within a certain distance threshold (8Å) are grouped together as a cluster. Then, each cluster is ranked based on a scoring function that is the sum of the z-scores of the pocket sites in a cluster.

Based on the benchmarking on various datasets 48 unbound/bound and 210 bound proteins, MetaPocket achieved slightly better results than the other approaches and correctly predicts the ligand the best overall success rate for all the top three predictions compared to each of the individual methods.

## Availability of the methods

In this section, we will describe the availability of these methods. Some methods are freely available for academic users as stand-alone version or some as web-servers and some as both. Table 1 lists the methods, their category, the URL where these methods can be accessed at and some important features of for the protein functional site prediction methods discussed in the paper.

**Table 1:** Availability, features and category of the computational functional site prediction tools discussed in this work.

Name	Category	URL	Features
Evolutionary Trace	Sequence-and-structure based	http://mammoth.bcm.tmc.edu/ETserver.html	Web-server, also available as Stand-alone version
ConSurf	Sequence, Sequence + structure	http://consurf.tau.ac.il	Web-server
MINER	Sequence	http://miniminer.sourceforge.net	Stand-alone version
FREPS (DSPAC index)	Sequence-and-structure based	http://freps.cbrc.jp	Appropriate Selection of homologous sequences, Web-server
ProBis	Structure based	http://probis.cmm.ki.si	Local structure alignment + geometry, Web-server + Stand-alone
FINDSITE	Structure based	http://cssb.biology.gatech.edu/findsite	Global structure alignment, Web-server
COFACTOR	Structure based	http://zhanglab.ccmb.med.umich.edu/COFACTOR/	Global + local structure alignment, web-server
LIGSITE	Structure-based	http://projects.biotec.tu-dresden.de/pocket	Geometric, Superseded by LIGSITE^csc^ server
LIGSITE^csc^	Structure-based	http://projects.biotec.tu-dresden.de/pocket/	Geometric, Web-server + stand alone
SURFNET	Structure-based	http://www.ebi.ac.uk/thornton-srv/software/SURFNET/	Geomertric, Stand-alone
Q-SiteFinder	Structure-based	http://www.modelling.leeds.ac.uk/qsitefinder/	Energetic, Web-server
Pocket-Finder	Structure-based	http://www.modelling.leeds.ac.uk/pocketfinder/	Energetic, Web-server
FOD	Structure-based	http://bioinformatics.cm-uj.krakow.pl/activesite/	Energetic, Web-server
FTSite	Structure-based	http://ftsite.bu.edu	Energetic, Web-server
ConCavity	Sequence-and-structure based	http://compbio.cs.princeton.edu/concavity/	Web-server + Stand-alone
SURFNET-ConSurf	Sequence-and-structure-based	Not available	Global + local structure alignement
MetaPocket	Structure-based	http://projects.biotec.tu-dresden.de/pocket/	Meta-approach, Web-server

## Summary and Outlook

There are a myriad of approaches for protein functional site prediction. Most of these methods can be broadly classified into sequence-based, structure-based or sequence and structure based approaches. Here, we mostly discussed sequence-and-structure based approaches. As discussed earlier, protein functional site prediction is a very hot topic in structural bioinformatics and we will continually see a lot of algorithms developed in the area. Especially, we might see more development of meta-server type of approach combining more than one methods and methods that combine both sequence and structure features. Furthermore, as protein structure prediction protocols are becoming mature, we will also see method development for prediction of protein functional sites for ‘modeled proteins’.

Moreover, most of these approaches use conservation as one of the important factor in determining the functional sites but these properties might be conserved because of the structural as well as functional reasons. Hence, methods like [50, 51] to distinguish sites that are conserved just due to structural reasons from the sites that are conserved due to functional reasons are needed to be developed in order to reduce false positives.
